# Loss-of-function *SLC30A2* mutants are associated with gut dysbiosis and alterations in intestinal gene expression in preterm infants

**DOI:** 10.1080/19490976.2021.2014739

**Published:** 2021-12-29

**Authors:** Shannon L Kelleher, Samina Alam, Olivia C Rivera, Shiran Barber-Zucker, Raz Zarivach, Takumi Wagatsuma, Taiho Kambe, David I Soybel, Justin Wright, Regina Lamendella

**Affiliations:** a Department of Cellular and Molecular Physiology, Penn State Hershey College of Medicine, Hershey, Pennsylvania, USA; b Department of Pharmacology, Penn State Hershey College of Medicine, Hershey, Pennsylvania, USA; c Department of Surgery, Penn State Hershey College of Medicine, Hershey, Pennsylvania, USA; d Department of Biomedical and Nutritional Sciences, University of Massachusetts Lowell, Lowell, Massachusetts, USA; e Department of Life Sciences, The National Institute for Biotechnology in the Negev and Ilse Katz Institute for Nanoscale Science and Technology, Ben-Gurion University of the Negev, Beer Sheva, Israel; f The Division of Integrated Life Science, Graduate School of Biostudies, Kyoto University, Kyoto, Japan; g Department of Biology, Juniata College, Huntingdon, Pennsylvania, USA

**Keywords:** Zinc, ZnT2, SLC30A2, host–microbe interactions, microbiome, infancy

## Abstract

Loss of Paneth cell (PC) function is implicated in intestinal dysbiosis, mucosal inflammation, and numerous intestinal disorders, including necrotizing enterocolitis (NEC). Studies in mouse models show that zinc transporter ZnT2 (*SLC30A2*) is critical for PC function, playing a role in granule formation, secretion, and antimicrobial activity; however, no studies have investigated whether loss of ZnT2 function is associated with dysbiosis, mucosal inflammation, or intestinal dysfunction in humans. *SLC30A2* was sequenced in healthy preterm infants (26–37 wks; n = 75), and structural analysis and functional assays determined the impact of mutations. In human stool samples, 16S rRNA sequencing and RNAseq of bacterial and human transcripts were performed. Three ZnT2 variants were common (>5%) in this population: H^346^Q, *f* = 19%; L^293^R, *f* = 7%; and a previously identified compound substitution in Exon7, *f* = 16%). H^346^Q had no effect on ZnT2 function or beta-diversity. Exon7 impaired zinc transport and was associated with a fractured gut microbiome. Analysis of microbial pathways suggested diverse effects on nutrient metabolism, glycan biosynthesis and metabolism, and drug resistance, which were associated with increased expression of host genes involved in tissue remodeling. L^293^R caused profound ZnT2 dysfunction and was associated with overt gut dysbiosis. Microbial pathway analysis suggested effects on nucleotide, amino acid and vitamin metabolism, which were associated with the increased expression of host genes involved in inflammation and immune response. In addition, L^293^R was associated with reduced weight gain in the early postnatal period. This implicates ZnT2 as a novel modulator of mucosal homeostasis in humans and suggests that genetic variants in ZnT2 may affect the risk of mucosal inflammation and intestinal disease.

## Introduction

Paneth cells (PCs) are considered the “gatekeepers of health”, and PC defects have been implicated in intestinal dysbiosis and numerous gastrointestinal disorders including dysmotility, colon cancer, inflammatory bowel disease (IBD), and necrotizing enterocolitis (NEC) in preterm infants.^1^Paneth cells are highly specialized secretory cells in the crypts of Lieberkühn that release large granules containing a variety of antimicrobial proteins, enzymes, cytokines, and growth factors that regulate the intestinal stem cell niche, manage the intestinal microbiome, modulate mucosal inflammation, and facilitate host microbial defense. Paneth cell granules also contain a remarkably high amount of zinc. Several lines of evidence suggest granule zinc depletion is pathogenic: severe zinc deficiency in humans causes PC granule zinc depletion and dysfunction,^2^ which can be remediated by zinc supplementation.^2,3^ Disruption of cellular zinc homeostasis using the zinc-specific chelator dithizone depletes granule zinc,^4–6^ which selectively kills PCs^6^ and leads to mucosal inflammation and intestinal permeability^7^ in rodent models. Moreover, dithizone administration followed by *Klebsiella* infection is commonly used as a preclinical mouse model of NEC,^8^ implicating PC granule zinc depletion in the pathogenesis of this disease. Thus, the ability to accumulate zinc in PC granules is critical for managing the composition of the gut microbiome and maintaining gastrointestinal health.

We recently showed that the zinc transporter ZnT2 (*SLC30A2*) is responsible for PC granule zinc accumulation and that ZnT2 deletion in mice leads to defective granule formation, secretion, and antimicrobial activity, which in turn were associated with oxidative stress, loss of barrier function and alterations in the gut microbiome.^5^ Of note, in epithelial cells outside the gastrointestinal tract, it has been reported that non-synonymous genetic variants in *SLC30A2*, many of which led to pathological reductions in zinc transport, are common in humans.^9,10^ These considerations suggest that defects in ZnT2 may have important health implications on the gut microbiome and gastrointestinal health in humans and offer a hypothesis that genetic variation in ZnT2 is associated with intestinal dysbiosis and dysfunction. Herein, we report a novel link between loss-of-function ZnT2 variants, gut dysbiosis, and poor initial growth in preterm infants and suggest an important physiologic context whereby genetic factors may play a role in the development of intestinal mucosal injury during early post-natal life.

## Results

### Study population

In total, 81 preterm infants were enrolled in the study at the Penn State Hershey Medical Center (PSHMC) between January 2014 and July 2017. The mean post-menstrual age (PMA) was 31.8 ± 2.4 weeks, and the mean birth weight was 1.74 kg ± 0.5. Our participants were predominately “healthy” at birth due to the exclusion criteria and as demonstrated by a robust 5 min APGAR (Table 1). Standard-of-care in the neonatal intensive care unit (NICU) at PSHMC includes a short (48 h) course of antibiotics (ampicillin/gentamicin ± vancomycin) at birth and 69 infants (93%) received prophylactic antibiotics. The majority of infants (73%, n = 59) were mixed fed (human milk + preterm infant formula (Similac® NeoSure) +/- Similac® Human Milk Fortifier); 23% of infants (n = 19) were fed exclusively human milk; and 4% of infants (n = 3) were fed exclusively formula (Similac® NeoSure; Table 2). There was a significant difference in feeding mode between genotypes, such that a significantly greater number of infants with L^293^R were fed exclusively human milk (60%; *P* < .01), while a significantly greater number of infants with Exon7 were mixed fed (85%; *P* < .01).

**Table 1.** Demographic summary of all infants enrolled in the study (n = 81)

	Number (%)	Mean ± SD
Male gender	43 (53%)	
White/Non-Hispanic	67 (84%)	
Hispanic	13 (15%)	
Vaginal delivery	30 (37%)	
Post-menstrual age at birth (weeks)		1.8 ± 2.4
Birthweight (kg)		1.74 ± 0.5
5 min APGAR, median (IRQ)		8 (7–9)

Values are mean ± standard deviation.

**Table 2.** Feeding mode of infants enrolled in the study

Feed Type	Total (n = 81)^#^	Wild type (n = 35)	L^293^R (n = 5)	Exon 7 (n = 13)	H^346^Q (n = 14)
Exclusively Human Milk (%)	19 (23%)	7 (20%)	3 (60%)*	2 (15%)	3 (25%)
Mixed feed (%)	59 (73%)	26 (74%)	2 (40%)	11 (85%)**	11 (69%)
Exclusively Formula (%)	3 (4%)	2 (6%)	0	0	0

^#^14 infants had either a genetic variant in *SLC30A2* other than those listed, or high-quality DNA sequences were not obtained.

*Significant difference in the percent of infants with L^293^R fed exclusively human milk, *P* < 0.01

**Significant difference in the percent of infants with Exon7 that were mixed fed, *P* < 0.01

Similar to what we reported in mice,^5^ ZnT2 was detected in the crypts of Lieberkühn in the human terminal ileum (Supplementary Figure 1). Sequence analysis of the *SLC30A2* coding region was conducted and adequate DNA sequences were obtained from 75 infants. We detected non-synonymous heterozygous ZnT2 substitutions in 53% of participants. All variants identified are summarized in Table 3. Single substitutions in ZnT2 were detected in 28% of participants, and compound substitutions were detected in 7%. A region of exon 7 containing numerous compound substitutions between amino acids 288 and 315 that was previously identified (referred to as Exon7)^9^ was documented in 16% of participants. The secondary structure of ZnT2 protein indicating all amino acid substitutions identified in this study was visualized using Protter (https://wlab.ethz.ch/protter/start/) in Supplementary Figure 2. In addition to Exon7, two variants were detected with a frequency greater than 5% (L^293^R, *f* = 7%; H^346^Q, *f* = 19%). These two novel variants were confirmed by TA cloning as previously described^9^ (Supplementary Figure 3).

**Table 3.** Summary of *SLC30A2* variants detected in the participants where high-quality DNA sequences were obtained (n = 75)

Genotype	Frequency (%)
Wild-type (WT)	35/75 (47%)
*Single (28%)*	
L^293^R	5/75 (7%)
H^346^Q	14/75 (19%)
R^291^H	1/75 (1%)
A^11^D	1/75 (1%)
*Compound (7%)*	
V^313^G/H^346^Q	1/75 (1%)
T^288^S/H^346^Q	2/75 (3%)
K^12^N/G^13^S	1/75 (1%)
D^103^E/H^346^Q	1/75 (1%)
*Multi-compound (16%)*	
Exon7*	12/75 (16%)

*Exon 7 substitutions: T^288^S, L^293^R, A^310^K, L^311^V, T^312^K, V^313^G, A^314^P, Q^315^P

**Figure 1.**
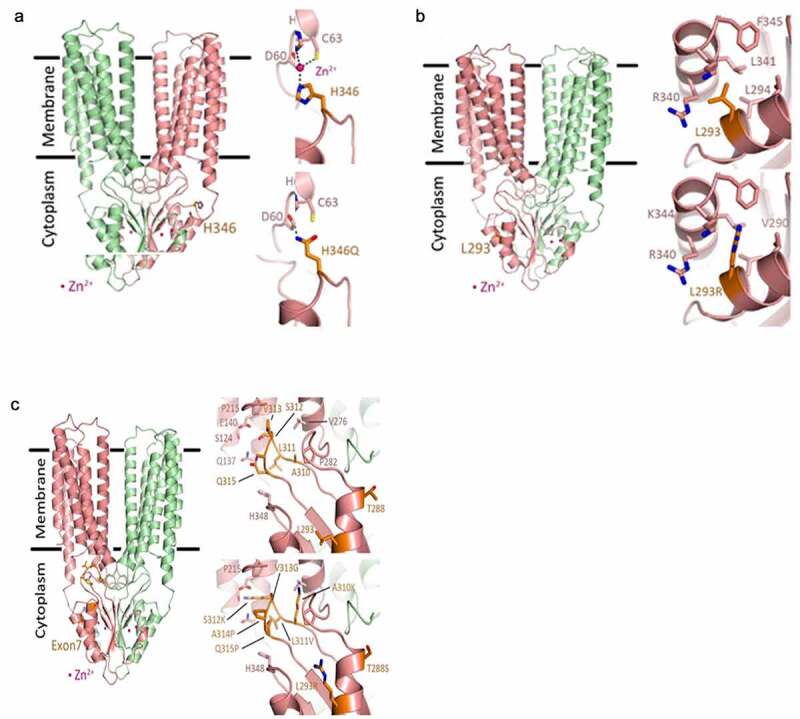
**Structural models of ZnT2**. The predicted dimeric human ZnT2 structure (chain A in salmon and chain B in pale green) based on human ZnT8 Cryo-EM structure (6XPD). The overall structure is in cartoon representation, the mutated residues are presented as orange sticks and the zinc cations are presented as pink spheres. In all panels, the Left image shows the mutated residues in the overall ZnT2 dimer structure. (a) H^346^Q. **Top right**, the wild type zoom-in view of H^346^ indicating possible zinc binding site with the predicted binding residues (represented as sticks) which lead to interaction of the CTD with the N-terminal tail. Dashed lines present the metal coordination interactions. **Bottom right**, H^346^Q might prevent zinc binding and can creates direct hydrogen bond (dashed line) between Q^346^ to H^62^. (b) L^293^R. **Top right**, the wild type zoom-in view of L^293^. L^293^ is located in a hydrophobic pocket between CTD α-helices α1 and α2. The interacting residues are represented as sticks. **Bottom right**, L^293^R might collide with F^345^, K^344^, and L^341^ thus will change the correct CTD fold due to insertion of positive charge into a hydrophobic pocket. (c) Exon7 (T^288^, L^293^, A^310^, L^311^, T^312^, V^313^, A^314^, Q^315^). **Top right**, the wild type zoom-in view of Exon7 residues (orange sticks) with the surrounding residues (pink sticks). Most of Exon7 residues form the β-hairpin loop between β1 to β2 strands (residues 310–315). This loop is positioned in a pocket formed by the transmembrane alpha helices which links the CTD and the transmembrane domains, creating the entrance to the cation transport channel (Q^315^ and S^312^). **Bottom right**, Exon7 mutations in the β-hairpin may collide with several residues and add positive charges into hydrophobic pocket (A^310^K) and into the cation entrance site in the transport tunnel (T^312^K). Additional mutations (V^313^G, A^314^P and Q^315^P) create a GPP sequence that will most likely prevent the formation of correct secondary structure fold that will prevent the C-terminal domain folding altogether. All these mutations will most likely cause a loss of function and prevent correct cation transport

### Structural modeling

Herein, we focused on the impact of the three most common variants with a frequency >5% (L^293^R, H^346^Q and Exon7). We first queried the impact of L^293^R, H^346^Q and Exon7 on ZnT2 structure. Structural models of ZnT2 were generated based on human ZnT8 (PDB:6XPD)^14^ (Figure 1). H^346^Q (Figure 1a): H^346^ sits on a connecting loop between helix 2 of the C-terminal domain (CTD) and the third CTD beta sheet. The loop is exposed to the solvent, but it faces the ZnT2 N-terminal tail prior to the first transmembrane alpha-helix. By examining the sequence and structure of this area, we noted three important amino acids (D,^15^ H^62^, and C^63^), all located in correct orientation such that they may create a zinc binding site between the N-terminal and the CTD. Base substitution at this site leading to a point mutation (H^346^Q) may prevent zinc binding and create a direct hydrogen bond; however, the effect of H^346^Q on zinc transport function was not readily apparent. L^293^R (Figure 1b): L^293^ is found between the two CTD helices (L^293^ in CTD-H1) in a hydrophobic pocket near the protein surface, and hydrophobic interactions between L^293^ to L^341^, R^340^, K^344^ and F^345^ (in CTD-H2) normally exist. The substitution of L^293^ to Arg can lead to collision or pi–pi stacking between R^293^-R^340^ or to charge repulsion from the approximate Lys and Arg residues. The former will serve to restrict the flexibility between the two helices such that the helices could bend toward or against each other. In the latter option, charge repulsion from the Lys residue will force the Arg to face toward the protein surface, and the overall interaction between the helices will decrease. As a result, the dynamics between the helices will be altered and the fold of the CTD will most likely be affected by disruption of the hydrophobic pocket. Furthermore, the addition of a positively charged residue (R^293^) could result in an even greater positive protein surface (dependent upon R^293^ orientation), due to the formation of region with a high density of positive charge. This likely affects the ability of L^293^R to bind zinc by the CTD domain, which would attenuate the zinc transport function. Exon7 (Figure 1c): The series of residues that comprise Exon7 form a β hairpin loop that is located at the entrance of the transmembrane metal transport site. In the outward facing state structure (6XPD), the Exon7 loop interacts with transmembrane helices (TM) 3,5, and 6 while in the inward facing state (6XPF) the Exon7 loop interacts only with TM 3 and 6. In general, the substitutions within the Exon7 loop change numerous hydrogen bonds and hydrophobic interactions in addition to potential loop disforming mutations, such as proline substitutions that change the loop conformation and polarity in the transmembrane metal transport site cytosolic entrance. Some key mutations in this region include Q^315^P and T^312^K. Q^315^P eliminates the polar residue glutamine in the metal transport site entrance, which might help with zinc entrance, and the addition of a proline might break the β-hairpin structure. T^312^K replaces a short polar non-charged residue with a large positively charged lysine. Such modification can modify the electrostatic potential of the pore and prevent zinc access to the transport site. In addition, the lysine might collide with TM 2 and 3 impairing the required conformational change between the outward and inward transmembrane domain conformations. The additional modifications such as A^310^K, V^313^G and A^314^P likely modify the β-hairpin structure due to possible collisions and addition of β-sheen destabilizing residues (Pro and Gly).

### Functional analysis of L^293^R, H^346^Q and Exon7

**Figure 2.**
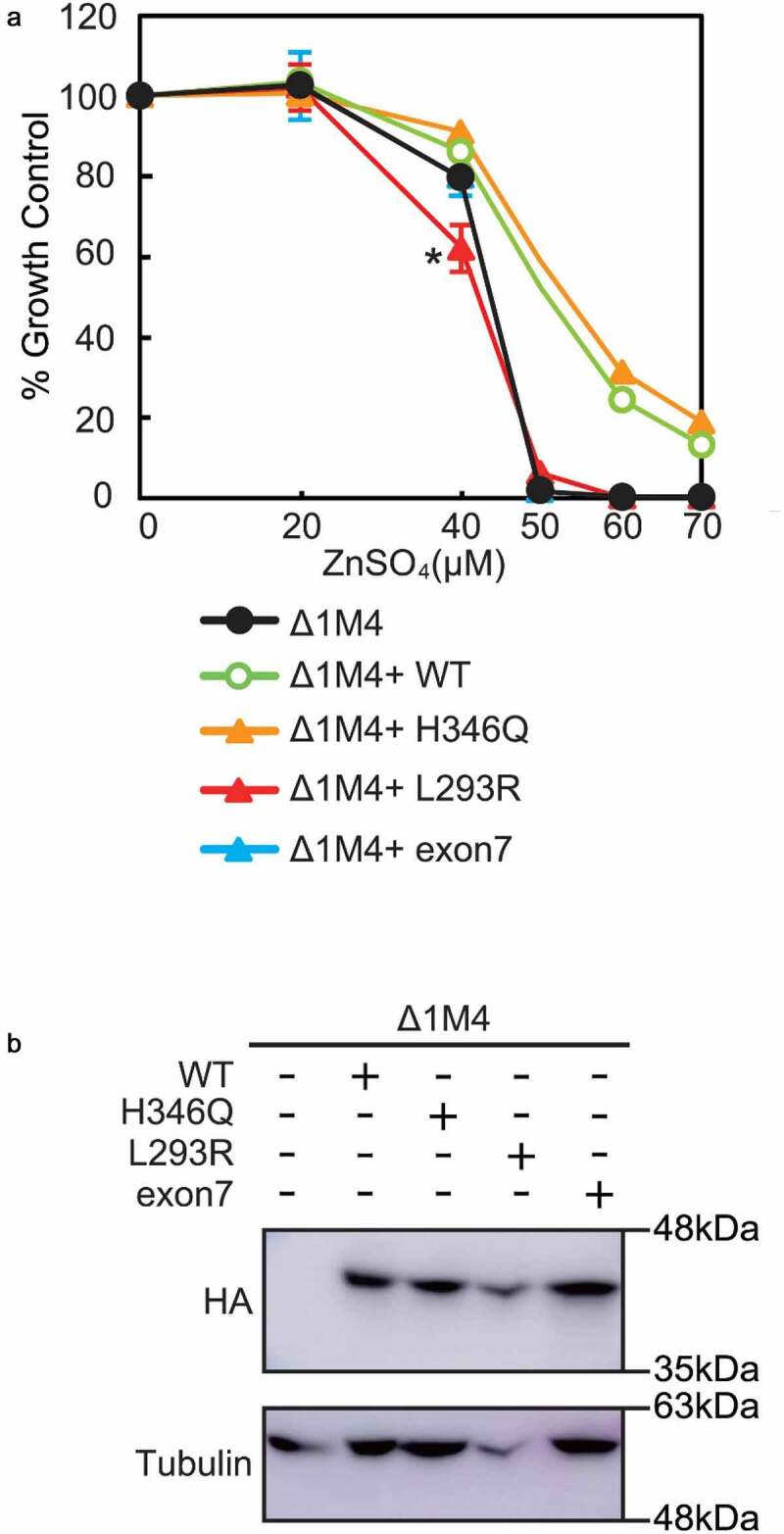
Cell-based zinc toxicity in DT40 cells stably transfected with various ZnT2 mutants. (a) The viability of cells exposed to the indicated concentrations of ZnSO_4_ for 48 h was determined by assessing the number of viable cells using alamar Blue. Data represent % growth relative to Δ1M4 cells ± SD, *P < .05 (data represent three independent experiments). (b) Representative immunoblots of cell extracts from Δ1M4 cells stably expressing WT ZnT2-HA and ZnT2-HA mutants used in A. Membranes were stripped and reprobed for β-tubulin as a loading control

To determine if these substitutions affected zinc transport activity, we used a functional assay in DT40 cells engineered to not express *ZnT1, MT* or *ZnT4* (Δ1M4 cells). As previously described,^16^ without the ability to export (ZnT1) or sequester (ZnT4 and MT) zinc, Δ1M4 cells cannot survive in the presence of >50 µM ZnSO_4_ unless ZnT2 is functional (Figure 2a). We expressed WT ZnT2 and L^293^R, H^346^Q and Exon7 mutants in Δ1M4 cells (Figure 2b) and compared cell growth as a readout of zinc transport activity. Expression of WT ZnT2 (Δ1M4+ WT) and the H^346^Q mutant clearly reversed the zinc-sensitive phenotype. In contrast, expression of the L^293^R and Exon7 mutants failed to do so (Figure 2). Moreover, we noted that Δ1M4 cells expressing L^293^R were consistently and significantly (*P* < .05) more sensitive to zinc than parent Δ1M4 cells, suggesting that expression of the L^293^R mutant has additional pathological consequences on cellular function. These results are consistent with our modeling predictions, and suggest that while H^346^Q can transport zinc, both Exon7 and L^293^R impair normal zinc transport.

### Microbiome analysis

**Figure 3.**
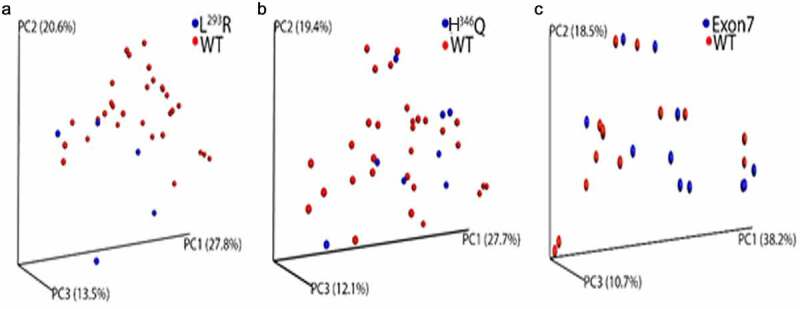
**Beta diversity of gut microbiome within L^293^R, H^346^Q, Exon7 and WT sample cohorts**. Principal-coordinate analysis (PCoA) plot generated using weighted UniFrac distances from a cumulative sum scaling (CSS)-normalized OTU table. (a) Significant differences in clustering were observed between L^293^R (blue) and WT (red) cohorts (ANOSIM *P* = .008). (b) No difference in clustering was observed when comparing H^346^Q (blue) and WT (red) cohorts. (c) A trend toward different clustering was observed between Exon7 (blue) and WT (red) cohorts, (ANOSIM *P* = .070)

High-quality 16S rRNA sequence data was obtained for 73/75 fecal samples, which were incorporated into the bioinformatics pipeline. Overall, sequencing depth of quality-filtered data ranged from 1,145 to 261,683 sequences/sample. Samples with a sequencing depth less than 1000 sequences were eliminated from the analysis. There was no effect of mode of delivery, early antibiotic administration, or infant diet on alpha or beta diversity (Table 4). In contrast, infants with Exon7 had a significant increase in observed species richness (alpha diversity, *P* = .01) compared to infants with two contiguous alleles (WT), while no significant differences were observed when comparing infants with L^293^R or H^346^Q to WT infants (Supplemental Figure 4). We next sought to determine if harboring H^346^Q, L^293^R, or Exon7 was associated with differences in microbiota composition. Weighted UniFrac (distance metric for comparing biological communities) principal coordinates analysis (PCoA) plots of WT infants compared to those with Exon7, L^293^R, or H^346^Q was conducted (Figure 3). There was no significant difference in Weighted UniFrac between infants with H^346^Q and WT ZnT2 (ANISOM *P* = .424), consistent with the lack of effect of H^346^Q on ZnT2 function *in vitro*. However, weighted UniFrac PCoA plots comparing WT infants and infants with Exon7 approached significance (ANOSIM *P* = .070) and were significantly different in infants with L^293^R (ANOSIM *P* = .008). Collectively, these data serve as a preliminary indication that defects in ZnT2 function led to different microbial communities in the gut.

**Table 4.** Effect of mode of delivery, maternal antibiotics and infant feed on Alpha and Beta diversity

Variable	Alpha Diversity (*P*-value)	Beta Diversity (Anosim) *P*-value
Mode of Delivery	0.295	0.876
Maternal antibiotics	0.209	0.641
Infant feed*		0.187
*Mother’s milk*	0.863	
*Donor Milk*	0.135	
*Human milk fortifier*	0.094	
*Formula*	0.739	

* Pairwise comparisons were made between the presence/absence of mother’s milk, donor milk, human milk fortifier or formula in the participant’s diet on Alpha diversity.

**Figure 4.**
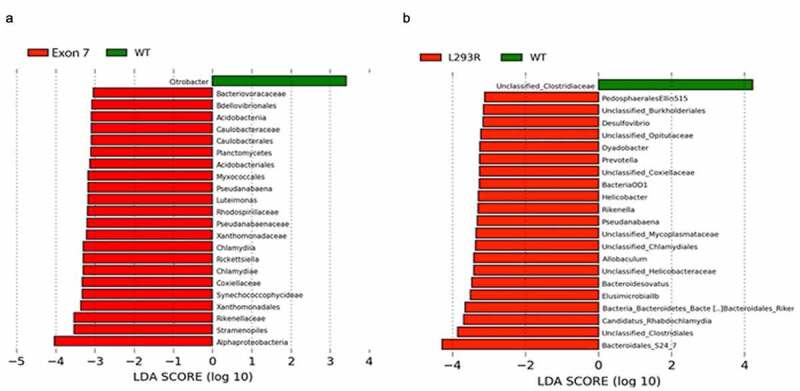
**Linear discriminant analysis effect size (LEfSe) plots display significantly differential taxa between** (a) **WT (green) and Exon7 (red) cohorts, and** (b) **WT (green) and L^293^R (red) cohorts**. Differential features with an LDA > 2.0 and *P* < .05 are considered significantly different

We next sought to determine differences in microbial genera in infants with L^293^R or Exon7. Initially, the infant gut microbiota is dominated by commensals such as *Bifidobacteria, Enterobacteriaceae*, and *Bacteroides*, and after weaning, bacterial orders such as *Clostridiales* and *Bacteroidetes* become more dominant.^17^ Pairwise LEfSE analysis revealed significant enrichment (LDA > 2.0, *P* < .05) of over 20 unique microbial taxa in the L^293^R and Exon7 cohorts when compared to WT infants (Figure 4). Of the 22 phylotypes enriched in infants with Exon7, few were recognized as commensal gut microbes (Figure 4a). Phylotypes enriched in infants with Exon7 included *Alphaproteobacteria* (particularly *Chlamydia* and *Rickettsia*), which are obligate intracellular pathogens. In addition, *Stramenopiles*, a complex assemblage of heterotrophic and photosynthetic protozoa including parasites such as *Blastocystis,* was one of the phylotypes most enriched in infants with Exon7. In contrast, *Clostridiales* and *Bacteroidales S24-7* were among the taxa most strongly enriched in infants with L^293^R when compared to WT infants (Figure 4b). In addition, infants with L^293^R were also enriched in *Helicobacter, Prevotella, Allobaculum*, and C*andidatus rhabdochlamydia. Helicobacter* are generally found in the stomach and various non-pylori *Helicobacter* spp. have been associated with IBD,^18^ enrichment of *Prevotella* has recently been implicated in gut inflammation,^19^ and *Rhabdochlamydia* has been detected in preterm infants and associated with more severe respiratory disease, need for mechanical ventilation and greater mortality.^20^ Collectively, loss of ZnT2 function is associated with physiologically deleterious changes in the gut microbiome, which may ultimately affect susceptibility to infection, mucosal inflammation, and disease.

**Figure 5.**
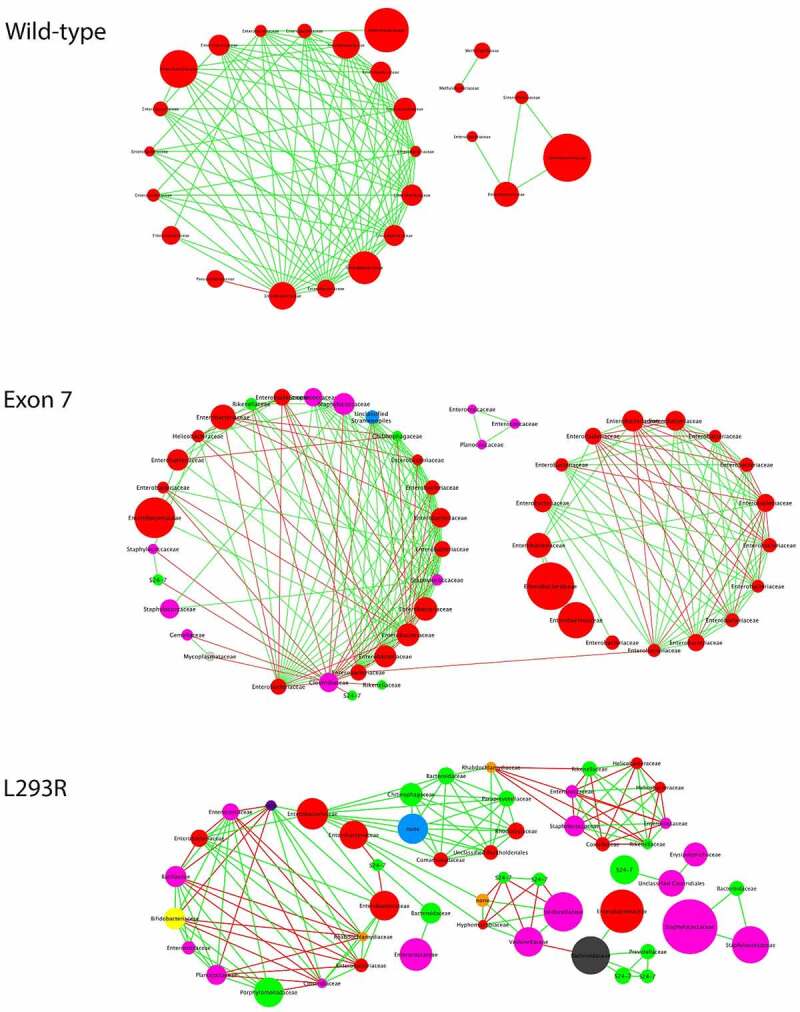
**Co-occurrence network plots**. Bipartite co-occurrence network plot of bacterial taxa within the fecal microbiome of participants with two contiguous ZnT2 alleles (WT), Exon7, or L^293^R. The co-occurrence network plot generated within the Cytoscape plugin Conet revealed strong positive (green) and negative correlations (red) between summarized OTUs. Summarized OUT nodes are colored by respective phyla

We next used a systems-oriented, graphical co-occurrence network analysis to enhance our understanding of the complex ecological structure and function and to illustrate differences in interrelationships between taxa in WT, and Exon7 or L^293^R cohorts (Figure 5). This methodology permits discrimination between a healthy gut microbiome and gut dysbiosis. In infants with two contiguous alleles, the microbial co-occurrence network plot clearly demonstrated the interdependence between taxa within a healthy gut microbiome. Note the predominance of *Enterobacteriaceae* as the dominant node and the co-exclusion of *Pseudomonadaceae*. By comparison, the microbial network plot of infants with Exon7 illustrates moderate disconnect between taxa within the gut microbiome, highlighted by the existence of two distinct clusters, one of which is dominated by *Enterobacteriaeae*, and a second cluster reflecting the overall importance of *Staphphylococcaceae* and *Clostridiaceae* to the network. In contrast, the microbial co-occurrence network plot of infants with L^293^R documents a profound disconnect in the interrelationships between taxa within the gut microbiome, illustrated by at least four distinct clusters. The largest cluster dominated by *Enterobacteriaeae, Porphyromonadaceae*, as well as numerous other taxa that facilitate both co-occurrence and co-exclusion correlations. One smaller cluster documented the importance of co-occurrence dominated by *Chitinophagaceae, Bacteriodaceae* and *Paraprevotellaeae*, a second small cluster dominated by *Staphphylococcaceae, Helicobacteraceae* and *Entreococcaceae* documenting both co-occurrence and co-exclusion, and a third small cluster dominated by *Veillonellaceae* and the exclusion of *Bacteriodales S24-7*. Finally, a disconnected “cluster” dominated by *Staphphylococcaceae* that co-occurred with *Bacteriodaceae, Enterobacteriaeae, Bacteriodales S24-7* and *Clostridiales* underpinned the microbiome. The abundance of *Staphphylococcaceae* and *Clostridiales*, which is associated with NEC,^21^ combined with the profound lack of interconnection and healthy co-occurrence, provides compelling evidence that harboring L^293^R leads to profound gut dysbiosis and may have consequences on gastrointestinal health.

### Metatranscriptomics

**Figure 6.**
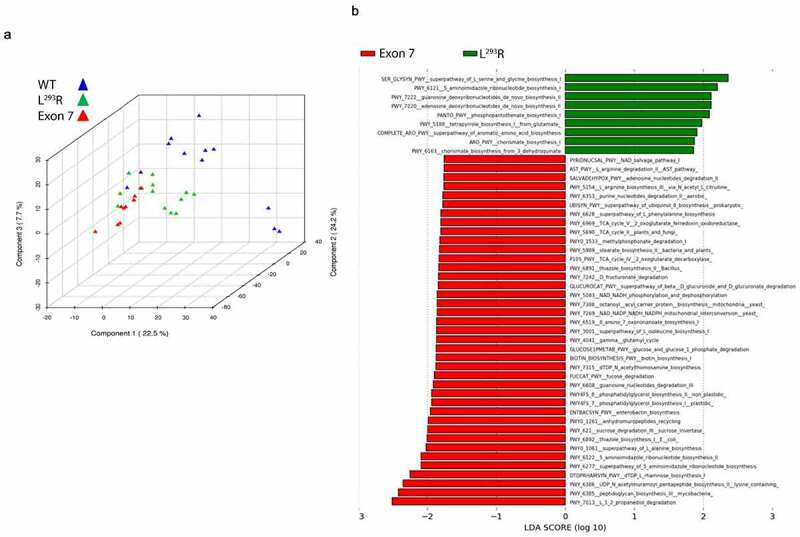
**Partial Least Squares Discriminant Analysis (PLS-DA) and LEfSe analysis of metatranscriptomic functional gene expression data**. (a) Partial least squares discriminant analysis (PLS-DA) was conducted within the METAGENASSIST data analysispackage utilizing a CPM normalized KEGG Orthology (KO) counts table of identified genes. (b) A LEfSe plot of significantly different functional gene Metacyc pathways (LDA > 1.0, *P* < .05) between Exon7 (red) and L^293^R (green) cohorts. Zero pathways were found to be overexpressed within the WT cohort, whereas 38 and 9 pathways were found to be enriched within the Exon7, and L^293^R cohorts, respectively

To assess the molecular activity of the microbial community, we analyzed the metatranscriptomic profiles of infants with L^293^R or Exon7 and compared them to infants with two contiguous ZnT2 alleles. KEGG Orthology comparative analysis was then conducted to compare functional molecular pathways between the three genotypes. Analysis of partial least squares discriminant (PLS-DA) plots clustered samples based on the differences in overall gene expression profiles (Figure 6a). Here, we observed distinct clustering indicating that a defined microbial gene expression profile exists within each respective genotype. Next, Metacyc functional pathway analysis compared differences in functional pathways between genotypes. LefSe plots were generated to identify significantly overexpressed pathways within each genotype (Figure 6b). In infants with Exon7, 39 pathways representing diverse categories were significantly overexpressed and yielded a linear discrimination analysis (LDA) score > 2.0; including two pathways associated with *carbohydrate metabolism* (propanediol degradation, sucrose degradation), *glycan biosynthesis and metabolism* (peptidoglycan biosynthesis, UDP Nacetylmuramoyl pentapeptide biosynthesis), and *nucleotide metabolism* (aminoimidazole ribonucleotide biosynthesis I & II), and one pathway associated with *amino acid metabolism* (alanine biosynthesis), *vitamin metabolism* (thiazole biosynthesis), *terpenoid and polyketide metabolism* (L-rhamose biosynthesis), and *drug resistance* (anhydromuropeptide recycling). Additional pathways of interest were overexpressed at an LDA < 2.0 within Exon7 including enterobactin biosynthesis (*terpenoid and polyketide metabolism)*, fucose degradation (*carbohydrate metabolism*), sterate biosynthesis (*lipid metabolism)*, and arginine biosynthesis (*amino acid metabolism*). In contrast, only nine pathways were significantly overexpressed in infants with L^293^R compared with infants with two contiguous ZnT2 alleles. Five pathways yielded an LDA score > 2.0 and included three pathways associated with *nucleotide metabolism* (aminoimidizole ribonucleotide biosynthesis, guanosine deoxyribonucleotide biosynthesis, adenosine deoxyribonucleotide biosynthesis), and one associated with *amino acid metabolism* (serine and glycine biosynthesis) and *vitamin metabolism* (phosphopantothenate biosynthesis).

### Host gene expression

**Figure 7.**
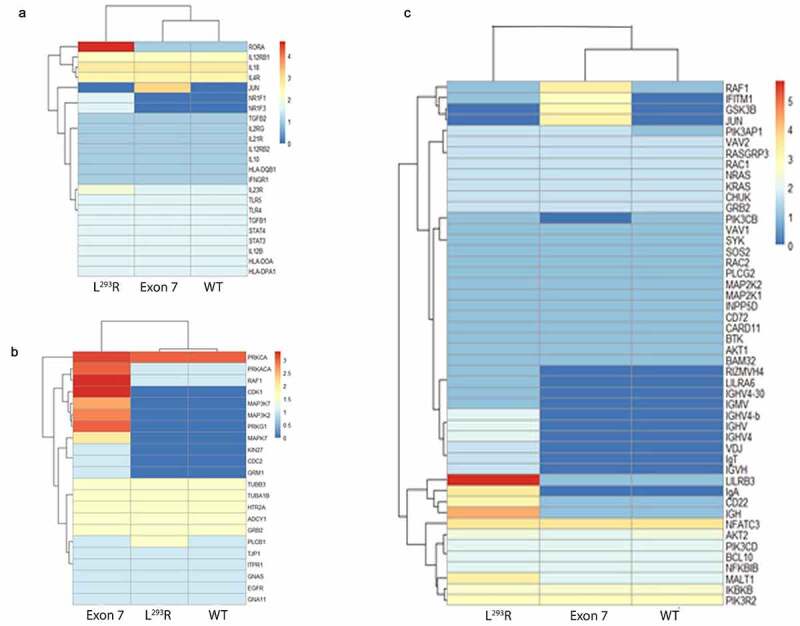
**Heatmaps of *Homo sapien* RNA-Seq functional gene annotations mapped to** (a) **Inflammatory Bowel Disease (IBD) KEGG pathway**, (b) **Gap junction KEGG pathway, and** (c) **B cell-associated KEGG pathway**. Homo sapien reads were annotated and extracted from the metatranscriptomic datasets. Assemblies were conducted on the host associated reads using MEGAHIT and EMBOSS was utilized to identify and extract open reading frames (ORFs) within the assemblies. Rstudio and the “pheatmap” package were used in the visualization of the heatmaps, with red indicating a higher number of samples yielding expression of the respective gene, whereas blue indicates a decreased number of samples yielding expression of the respective gene

We next conducted an analysis to identify potential effects of loss of ZnT2 function on intestinal gene expression. We took advantage of the presence of metatranscriptome reads identified as “*Homo sapien*” by KneadData and subsequently used them for functional host gene annotation (Figure 7), also known as the exfoliome.^22^ Attempts to map genes to Paneth cell-specific pathways were unsuccessful as we were limited in the information we were able to extract from the metatranscriptomic dataset due to the sequencing depth of the *Homo sapien* dataset and this level of granular detail is a limitation of the KEGG database. In addition, the plausibility of obtaining meaningful data reflecting changes in the expression of Paneth cell genes using the exfoliome is very low due to the biology of these long-lived, terminally differentiated cells that reside in the crypts of Lieberkühn of the ileum. Therefore, we selected three functional pathways that were of specific biological interest (B-cell function, gap junction, and IBD pathways) and also because they each yielded multiple functional gene hits. Genes were organized into clusters of specific interest: “IBD-associated genes” (Figure 7a), “Gap junction-associated genes” (Figure 7b) and “B cell-associated genes” (Figure 7c). Infants with Exon7 had greater expression of genes within the “Gap junction-associated genes” cluster including *PRKCA/KIN27, RAF1, CDK1/CDC2, MAPKs*, and *GRM1* (Figure 7b). This expression profile infers that metabolic consequences of harboring Exon7 lead to a “remodeling” phenotype in the gut. In contrast, infants with L^293^R had greater abundance of genes in both the “IBD-associated genes” cluster including *RORA/NR1F1, NR1F3, IL23R*, and *PLCB1* (Figure 7a), and “B cell-associated genes” cluster including *IGHV/VDJ, LILRB3, IgA, Cdc22*, and *MALT1* (Figure 7c). Collectively, this expression profile suggests that metabolic consequences of harboring L^293^R leads to an “inflammatory phenotype” in the gut.

### Infant health outcomes

Given the relationship between defects in PC function and NEC, our initial goal was to determine if genetic variants in ZnT2 were associated with NEC. However, major changes in standard-of-care policies implemented during our study period reduced the incidence of NEC from 12% to 4%, eliminating the statistical power to test our original hypothesis. However, because our study design eliminated unpredictable environmental and dietary influences (participants were housed in the same environment, cared for by the same team of providers, and fed a consistent diet from birth through the first two weeks of life), it provided a unique opportunity to determine the effects of ZnT2 function on the developing gut microbiome and intestinal response. Consistent with our prediction of an “inflammatory phenotype”, infants harboring L^293^R tended to gain less weight from birth through the first two weeks of life compared with WT infants (*P* = .08). Moreover, we noted that 60% of infants with L^293^R gained minimal weight (<0.5 g/d) over the course of the study (3/5 infants lost weight over the first two weeks of life), compared with 6% of WT (*P* < .001) or infants with H^346^Q (*P* < .01), and 8% of infants with Exon7 (*P* < .05). However, there were no differences in the number of infants who required an additional course of antibiotics, had suspected GI issues, or who were diagnosed with NEC between genotypes (Table 5).

**Table 5.** Infant health outcomes

	Wild type (n = 35)	L^293^R (n = 5)	Exon 7 (n = 13)	H^346^Q (n = 16)	p-value
Mean weight gain, g/d	8.18 ± 5.12	3.34 ± 7.98*	5.61 ± 7.83	5.79 ± 4.02	*P = .08
Gain < 0.5 g/d (%)	6% (n = 2)	60% (n = 3)*	8% (n = 1)	6% (n = 1)	*P < .01
Prolonged antibiotic exposure	14% (n = 5)	0	23% (n = 3)	6% (n = 1)	
Suspected GI issues	17% (n = 6)	0	23% (n = 3)	6% (n = 1)	
NEC diagnosis	6% (n = 2)	0	8% (n = 1)	0	

## Discussion

Herein, we detected three variants in ZnT2 with >5% frequency in preterm infants, characterized their structure–function relationships and determined the consequences of harboring these variants on the developing gut microbiome, intestinal gene expression, and several predictive health outcomes.

While complete loss of ZnT2 function in ZnT2-null mice leads to gut dysbiosis, oxidative stress, loss of barrier function, and an exaggerated inflammatory response,^5^ consistent with previous reports, not all non-synonymous substitutions in ZnT2 led to defects in zinc transport.^10^ The most common variant identified in our participants (H^346^Q; *f* = 19%) had no effect on ZnT2 function or beta diversity of the gut microbiome. Structural modeling predicted that H^346^ is exposed and theoretically might interact with the unstructured N-terminus of ZnT2. Thus, we cannot exclude the possibility of a subtle and less apparent pathologic alteration in ZnT2 function or post-translational regulation. In contrast, our study provides compelling evidence that while the other two variants (Exon7 and L^293^R) both impaired ZnT2 function, they lead to profoundly different consequences on the gut microbiome and host GI response.

Consistent with our structural model predicting potential alterations in the energetic cost needed to transition between open and closed conformations reducing zinc transport, functional analysis of Exon7 suggests zinc transport activity was impaired and a modest shift in microbial connectivity and stability was observed. Two distinct microbial networks were identified; one was dominated by members of commensal *Enterobacteriaceae*, and the other comprised of greater diversity and numerous taxa not generally recognized as commensal gut microbes. Several taxa were identified that may have adverse consequences on health. In particular, *Chlamydia* and *Rickettsia*, which are obligate intracellular pathogens, were significantly enriched. *Chlamydia* is believed to infect via the fecal-oral route, while *Rickettsia* is a natural parasite of arthropods (i.e., fleas, ticks, mites, lice). Additionally, enrichment of *Stramenopiles*, a complex assemblage of heterotrophic and photosynthetic protozoa that include parasites, such as *Blastocystis*, was identified. *Blastocystis* is common in developing countries^23^ and is associated with diarrhea, abdominal pain, and vomiting, but its role as a causative agent in human diseases is unclear. *Xanthomonodales* are multidrug opportunistic pathogens that are responsible for nosocomial infections and were also profoundly enriched. Collectively, this suggests harboring Exon7 may increase vulnerability to nosocomial GI infections.

This altered microbial environment may stem from defects in PC function and/or granule activity. Loss of ZnT2 function impairs zinc transport into PC granules, which reduces lysozyme function and antibacterial activity in ZnT2-null mice.^5^ Key antibacterials such as lysozyme play a significant role in defense against bacterial pathogens and in regulating the interactions between gut microbiota and host immune systems, consistent with changes in the structure of the gut bacterial community we detected. Alternatively, previous studies found Exon7 is associated with significantly greater zinc secretion,^9^ most likely stemming from compensatory zinc transport mechanisms. Although luminal zinc concentrations were not measured in this study, we speculate Exon7 may lead to greater zinc secretion into the GI lumen, which could alter the gut microbiome. In support of this possibility, several reports directly link excess zinc exposure to increased *Chlamydia* growth and infection,^24^ and enriched *Xanthomonodales* abundance in sedimentary ecosystems.^25^ Moreover, excess zinc exposure alters the gut microbiome^26^ in piglets, and causes dysbiosis^27^ in mice. In addition, we previously showed excess dietary zinc significantly enriches *Strameopiles* and *Rikenellaceae* in mice,^27^ similar to infants harboring Exon7. However, whether excess zinc is secreted into the gut lumen in infants harboring Exon7, and whether it is substantial enough to alter the gut microbiome requires further investigation.

Despite enrichment of numerous non-commensals, infants harboring Exon7 had a high level of diverse metabolic activity, which may actually have positive consequences on the host. Recent studies implicate propanediol, fucose and rhamnose as cross-feeding metabolites that modulate trophic interactions between symbionts,^28^ which may serve to promote stability of the microbiome. Additionally, these metabolites may have positive consequences in preterm infants. For example, propionate is taken up by colonocytes^29^ and used in gluconeogenesis in the liver,^30^ which is particularly important to glucose regulation in preterm infants.^31^ Sterate is used for fatty acid biosynthesis and can activate AP1^11^ increasing expression of MUC2^32^ and production of the protective mucus layer in the gut. One of the most significantly enriched microbial genes was formylCoA transferase (LDA = 2.17, *p* = 1.19^-5; Supplementary Table 1), an oxalate degrading enzyme associated with protection from calcium oxalate urolithiassis.^33^ Preterm infants are at the risk of development of nephrocalcinosis,^34^ and thus, enhanced degradation of oxalates may be beneficial. Another interesting gene of note is β-mannosidase (LDA = 3.28, *p* = 3.5^-5). Mannose is particularly important for infant growth, and free mannose, in addition to mannose in the form of oligosaccharides, is present in high concentrations in breast milk,^35^ contributes to the establishment of nonpathogenic colonic flora, inhibits binding of pathogens to the intestinal epithelial cell,^36^ and improves growth in weanling pigs.^37^ This suggests harboring Exon7 may provide some benefit to the host. Consistent with a potential positive impact, Exon7 was associated with upregulation of numerous host genes that play roles in proliferation, differentiation, migration, and adhesion (*JUN, RAF1, PRKCA/KIN27* and *MAPKs*), cell cycle (*CDK1/CDC2*), and calcium signaling (*GRM1*) in the gut. Moreover, expression of *GSK3b*, critical for Wnt3 signaling, was increased, which may enhance communication between PCs and the intestinal stem cell niche.^38^ In sum, this expression profile combined with expression of key metabolites suggests infants with Exon7 may be responding to cues that activate tissue remodeling and promoting development of the gut.

In contrast, L^293^R may have profound negative consequences on the host. Consistent with the predicted changes in the dynamics between the two C-terminal domain helices and the inability to bind zinc, expression of L^293^R significantly enhanced cellular zinc sensitivity. This could result in profound consequences on cellular function, such as defects in intracellular signaling, autophagy, or apoptosis.^39^ Moreover, these defects in PCs may affect intestinal stem cells and the maintenance of the entire intestinal environment,^40^ thereby increasing susceptibility to mucosal inflammation and disease. This may be due to localization of L^293^ within a unique regulatory domain. We recently reported that the unique L^293/294/295^ trileucine motif in ZnT2 responds to downstream de/phosphorylation motifs and is critical for adaptor-3 (AP3) binding, intracellular targeting of lysosomes, and activation of lysosomal mediated cell death.^39^ This suggests substitution of R^293^ may alter intracellular targeting and promote cell death pathways; however, further functional genomics studies are required to understand the impact of L^293^R in PCs. It is curious that the L^293^R substitution is found within the Exon7 variant, yet the consequences of L^293^R and Exon7 on ZnT2 function are different. Studies to systematically determine the contribution of each substitution on ZnT2 structure–function relationships are required to understand this observation.

Deleterious consequences of L^293^R on ZnT2 function led to profound disruption in network density, connectivity and stability within the gut microbiome, illustrating fractured interrelationships within the microbial community. The gut microbiome was dominated by eight different families, organized into four minor yet distinct subcommunities, and this profound lack of connectivity and stability is the hallmark of gut dysbiosis. Interestingly, *Clostridiales* and *Bacteroidales S24-7* were among the taxa most strongly enriched in infants with L^293^R, consistent with our previous observations in ZnT2-null mice, where we also found that *Bacteroidales S24-7* was most enriched.^5^ Moreover, *Bacteroides ovatus*, a predominant commensal bacteria associated with antibody response in IBD, was enriched in infants harboring L^293^R, as was C*andidatus rhabdochlamydia*, which is associated with severe respiratory distress in premature infants.^41^

One of the most significantly enriched microbial gene within infants harboring L^293^R is a serine protease (Supplementary Table 1) and is of great interest, as increased expression is a response to inflammation, and deactivation of this gene is utilized as a therapeutic approach.^42^ Moreover, fecal serine protease is used in profiling IBD,^43^and Schlapbach and colleagues found increased blood levels of serine protease-2 in infants with NEC.^44^ In addition, nucleotide production was upregulated, which can be taken up by nucleoside transporters (*SLC28* and *SLC29* families) to support intestinal development and recovery and is used by the host to enhance mucosal repair, improve barrier function, and increase DNA and protein synthesis and cellular proliferation.^45^ Consistent with this observation, infants with L^293^R upregulated genes associated with inflammation and IBD, such as *RORA/NR1F1*, a downstream target of IL6 and TGFβ that regulates IL17 and Th17 lymphocyte production, and *NR1F3* and *IL23R*, which activate Th17. In addition, infants with L^293^R had greater expression of genes involved in immune response including *IGHV/VDJ, LILRB3, IgA, Cdc22*, and *MALT1*. Collectively, interpretation of this expression profile suggests infants with L^293^R may be robustly responding to mucosal inflammation. Most importantly, we found that infants harboring L^293^R tended on average to gain less weight in the first two weeks of life (*P* = .08) and was associated with a significantly greater number of infants who gained weight sub-optimally (< 0.5 g/d, *P* < .01) compared to the other genotypes. In fact, 60% of infants harboring L^293^R lost weight over the first two weeks of life, compared to 6–8% in infants with the other ZnT2 genotypes, suggesting harboring this ZnT2 variant may indeed have critical consequences on early growth and development.

There are several limitations of this study. Most importantly, this study was not designed to determine associations between genetic variation in ZnT2 on specific health outcomes in preterm infants, and we do not interpret these data to suggest that genetic variation in ZnT2 is associated with increased risk for disease. Our study does however provide compelling evidence that specific defects in ZnT2 function may have important consequences on the gut microbiome and intestinal health that warrant further investigation. Secondly, there was a significant difference in mode of feeding between genotypes. Interestingly, we noted that while harboring L^293^R was associated with profound gut dysbiosis 60% of these infants lost weight in the early postnatal period despite the fact that they were exclusively fed human milk (the remaining were mixed fed). It is intriguing that the higher prevalence of human milk feeding in this cohort did not provide protection. Third, the effect of L^293^R on weight gain should be interpreted with caution as there were only five infants with L^293^R and further studies are required to fully understand the relationship between this variant and early neonatal growth. In addition, it is important to note that this was an acute study and long-term health outcomes were not explored. Therefore, a larger prospective study designed to explore the effects of ZnT2 variants on both short- and long-term health outcomes is warranted.

In summary, our study provides compelling evidence of a novel link between ZnT2 and gut dysbiosis and health in humans and shows that loss-of-function ZnT2 variants have clear consequences on the developing gut microbiome and host–microbe interactions. The mechanism(s) by which ZnT2 dysfunction elicits these effects may open future directions of study regarding the pathogenesis and potential treatment of devastating diseases such as IBD and NEC.

## Methods

### Study population

A prospective study to determine the relationship between genetic variation in ZnT2 and host–microbe interactions was conducted at Penn State Hershey Medical Center (PSHMC) between January 2014 and July 2017. The study protocol was approved by the Institutional Review Board of the Pennsylvania State University. Inclusion criteria included infants born between 26 and 37 week gestation and admitted to the PSHMC neonatal intensive care unit (NICU) or transferred to the NICU within 72 h of birth. Exclusion criteria included infants born <26 or >37 wk gestation, born with major congenital anomalies (heart, gastrointestinal, renal, or respiratory tract), born to mothers known to use illicit drugs or abuse alcohol, or to mothers with a history of depression requiring long-term psychotropic medication. Written consent was obtained from all subjects. Metadata on infant feeding history and clinical course while in the NICU were collected electronically.

### Sample Collection

DNA was collected using a buccal swab and total genomic DNA was isolated (MasterAmp^TM^ Buccal Swab DNA Extraction Kit, Epicenter Biotechnologies). Fecal material was collected ~2 weeks after ~48 h of prophylactic antibiotics was discontinued (standard-of-care), and enteral feeds were initiated. Fecal material was transferred to a sterile microfuge tube immediately following defection and frozen at −80°C until analysis.

### SLC30A2 sequencing and confirmation

Genomic DNA (100 ng) was amplified by PCR using primers specific for exon, PCR products were purified, and variants were identified by direct sequencing using the 5′ primers used to amplify the respective exons as previously described.^46^ The chromatograms were base-called with Applied Biosystems Sequencing Analysis 5.2 Patch 2 software and visually inspected. PCR amplifications were replicated three times and were considered confirmed when nucleotide changes were reproduced in all independent PCR amplifications. Sequences were compared with the predicted protein sequence deposited in the National Center for Biotechnology Information database (http://www.ncbi.nlm.nih.gov/protein/NP_001004434.1). Novel ZnT2 variants were validated by TA cloning as previously described.^9^

### Structural modeling of ZnT2

The three-dimension model of ZnT2 was built using the SWISS-MODEL Automatic Modeling Model,^47–53^ based on the structure of human ZnT8 Cryo-EM structure (6XPD).^14^ Mutations have been implemented by Coot^54^ and structural model figures were prepared using the PyMOL Molecular Graphics System, Version 2.0 Schrödinger, LLC.

### Histology

Sections (5 µm) of human intestinal tissue were kindly provided by Drs. G Yochum and W Koltun (PSHMC) and randomly selected from the colorectal disease biobank supported by the Peter and Marsha Carlino Fund for IBD research (IRB #HY98-057EP-A; W Koltun, PI). Antigen retrieval was performed for 10 min using citrate buffer (Vector Laboratories). Sections were permeabilized with 0.2% Triton X-100 (Sigma-Aldrich) in PBS for 45 min and blocked in 10% donkey serum, 1% BSA, and 0.3% Triton X-100 in PBS for 1 h. Sections were incubated with ZnT2 primary antibody (1:1000; Biorbyt) overnight at 4°C and detected with Alexa Fluor 594 donkey anti-rabbit IgG (1:1,000; Invitrogen). Nuclei were stained with 4,6-diamidino-2-Phenylindole, dilactate (DAPI; 1:1000; Invitrogen). Images were collected with an inverted confocal microscope (Leica Microsystems, Model SP8).

### Plasmid construction

Mutant constructs were generated using the QuikChange II Site-Directed Mutagenesis Kit (Agilent Technologies) and mutations were confirmed using directed sequencing as previously described.^46^ For *in vitro* studies, a construct comprised of multiple substitutions found in Exon7 was generated (T^288^S/L^293^R/A^310^K/L^311^V/T^312^K/V^313^G/A^314^P/Q^315^P).

### Cell culture

DT40 cells deficient in *ZnT1, ZnT4*, and metallothionein (*MT*) genes (Δ1M4 cells) were generated previously.^16^ Wild-type DT40 cells and Δ1M4 cells were maintained in RPMI 1640 medium (Nacalai Tesque) supplemented with 10% heat inactivated fetal calf serum (Multiser, Trace Scientific Ltd.), 1% chicken serum (Invitrogen), and 50 μM 2-mercaptoethanol (Sigma-Aldrich) at 39.5°C, as described previously.^16^ Δ1M4 cells were stably transfected with plasmid containing C-terminally HA-tagged WT or mutant ZnT2 using electroporation.^55^ The transfected cells (10^5^ cells/mL) were seeded in 96-well plates and treated with 20–70 µM ZnSO_4_ for 2 d. Alamar Blue reagent (AbD Serotec, Ltd.) was added to the culture media for 4 h, and absorbance was determined at 570 and 600 nm (PowerScan 4; DS Pharma Biomedical).

### Immunoblotting

Immunoblotting was performed as described previously.^56^ Antibodies used were anti-HA (HA-11, 1:3000; BioLegend) and anti-α-tubulin (12G10, 1:3,000; Developmental Studies Hybridoma Bank (DSHB) by Frankel, J. and Nelsen, E.M.). Immobilon Western Chemiluminescent HRP Substrate (Millipore) was used for detection. The fluoroimage was obtained using ImageQuant LAS 500 (GE Healthcare).

### Fecal Microbiota Analysis by 16S Ribosomal RNA Sequencing

DNA extraction, 16S rRNA amplification, and high-throughput sequencing on the Illumina MiSeq were conducted by Wright Labs, LLC as previously described.^57^ Sequences were trimmed at 150 bp, quality filtered at an expected error of less than 0.5% using and analyzed using the QIIME 1.9.0 software package. Downstream chimera removal, alpha diversity, beta diversity, and LEfSe analyses were conducted as described previously.^57^

### Metatranscriptomic Analysis

RNA extraction and metatranscriptomic library preparation and Illumina sequencing were conducted on 0.25 g of fecal material by Wright Labs, LLC as previously described.^58^ Raw read quality was assessed using FastQC to obtain average Q scores across the read length of all R1 and R2 fastq files. Trimmomatic was used to quality filter and pair the raw sequence data.^59^ A sliding window filtration was utilized to cut reads at a 4-base average Q score of 28 or lower and reads trimmed below 60 basepairs were discarded. Filtered reads were paired, and all reads not successfully paired were discarded.

*Bacterial transcriptome* – Paired FASTQ were concatenated and run through the KneadData pipeline to remove host (human) DNA reads from filtered sequence data. MetaPhlan2 was implemented to quantify the active bacterial taxonomic profile within each sample.^12^ Taxonomic output from all samples was merged into a single biom file for downstream analyses. To obtain functional gene profiles, filtered data was annotated using the Uniref90 database within Humann.^13^ Uniref90 annotations were regrouped as Metacyc pathways and consequently underwent CPM (counts per million) normalization for LEfSe enrichment analysis, which was run with default settings.^60^ Comparisons were made with “genotype” as the main categorical variable (“Class”). Linear Discriminant Analysis (LDA) scores greater than 2.0 were used for pathway enrichment plotting.

*Human transcriptome* – Metatranscriptome reads identified as “*Homo sapien*” by kneaddata underwent functional gene annotation. Extracted human reads were assembled using MEGAHIT^15^ and EMBOSS was utilized to identify and extract open reading frames (ORFs) within the assemblies.^61^ Read annotation was preformed using tBLASTn. KEGG functional gene pathways of interest (IBD, gap junction and B-Cell) were constructed from NCBI’s GenBank database. Counts of presence and absence of genes from pathways of interest were determined from tBLASTn outputs. Hits with a bit score of 40 or higher were included in the analysis and heatmaps were generated using presence/absence counts from tBLASTn. Rstudio and the “pheatmap” package were used in the visualization of generated heatmaps (https://rdrr.io/cran/pheatmap/). Genes unable to be annotated from Genbank were excluded from heatmap plotting.

### Statistics

All data are reported as mean ± standard deviation. Statistical analysis for functional assays was conducted using Student’s t-test. Alpha diversity comparisons were conducted using a two-sample t-test and nonparametric Monte Carlo permutations (n = 999) within QIIME-1.9.1. Analysis of similarity (ANOSIM) tests for significance were calculated within QIIME 1.9.1 to determine the significance of clustering between disease cohorts. One way ANOVA was used to compare differences in feeding mode among all infants. Student’s t-test was used to compare mean weight gain during the first two weeks of life for infants with each ZnT2 variant with those harboring two contiguous alleles. Chi-square analysis was used to compare the number of infants with each ZnT2 variant who gained minimal weight (<0.5 g/d), had prolonged antibiotic exposure, or suspected GI issues during the first two weeks of life, or who had a diagnosis of NEC while in the NICU, with those harboring two contiguous alleles. Statistical analysis was conducted using GraphPad Prism (version 9.1.1) and a significant difference is reported as *P* < .05. All authors had access to the study data and had reviewed and approved the final manuscript.

## Supplementary Material

Supplemental Material

## Data Availability

The data that support the findings of this study are openly available in the BioProject database at http://www.ncbi.nlm.nih.gov/bioproject/736070, reference number PRJNA736070, and in the Figshare database at http://www.figshare.com/10.6084/m9.figshare.16692400.
