# Supplementary material for: Loss-of-function SLC30A2 mutants are associated with gut dysbiosis and alterations in intestinal gene expression in preterm infants
Source: Gut Microbes. 2021 Dec 29;14(1):2014739. doi: 10.1080/19490976.2021.2014739 (PMC8726655; doi:10.1080/19490976.2021.2014739)
Supplement: Supplemental Material [file KGMI_A_2014739_SM6752.zip › supplementary/downloadFromZipFile.pdf]

Supp Figure 1. ZnT2 in human terminal ileum

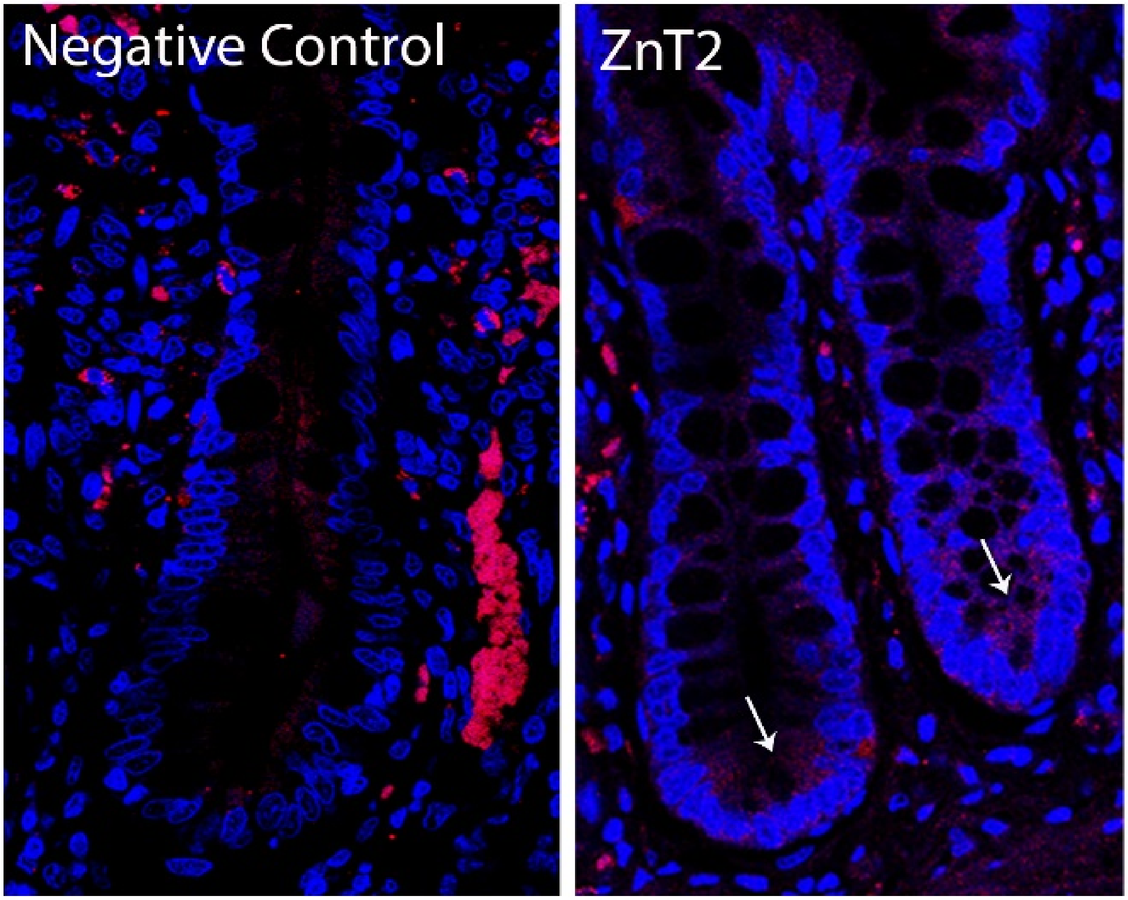

Supp Figure 2. Protter visualization of secondary structure

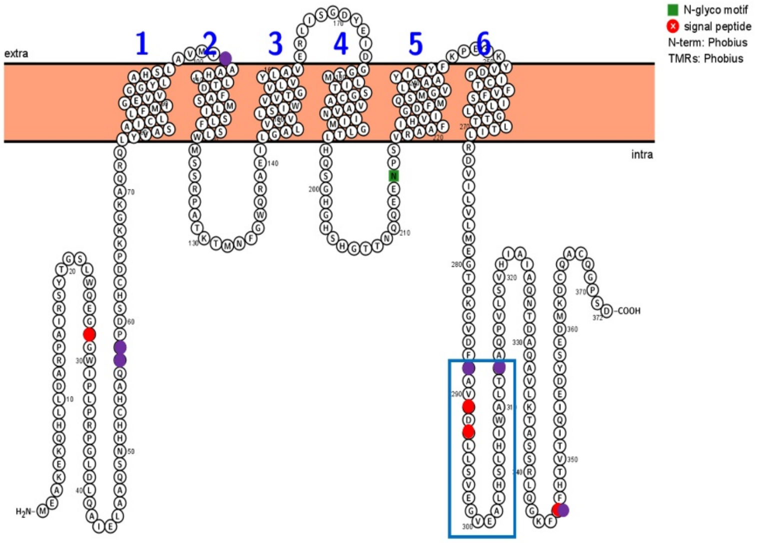

Supp Figure 3. Sequence confirmation of $L^{293}R$ and $H^{346}Q$

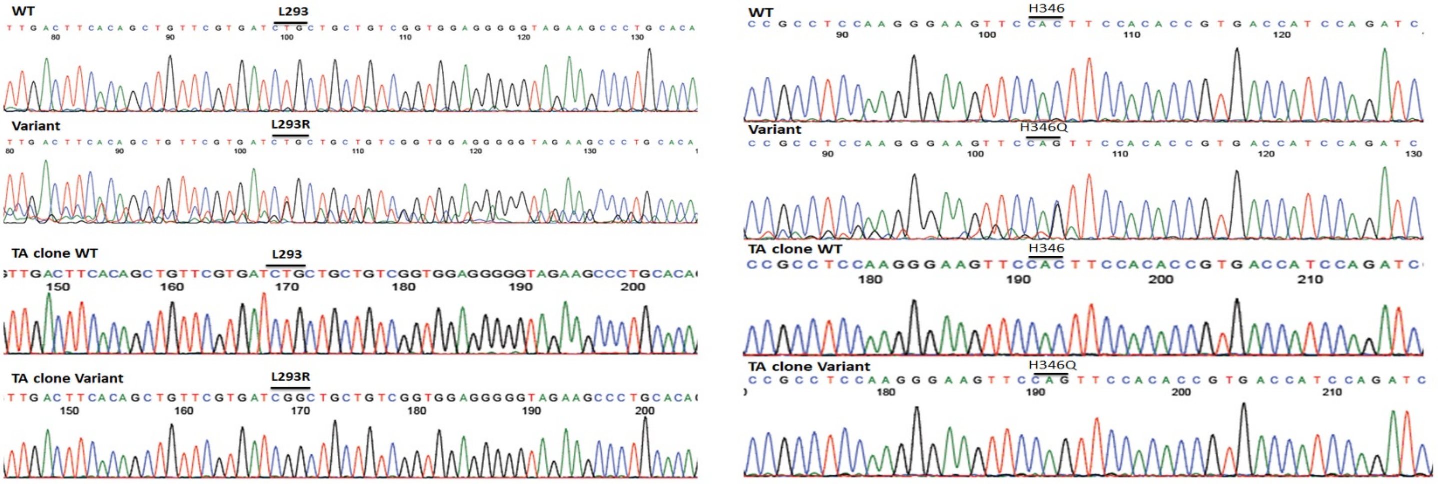

Supp Figure 4. Alpha diversity

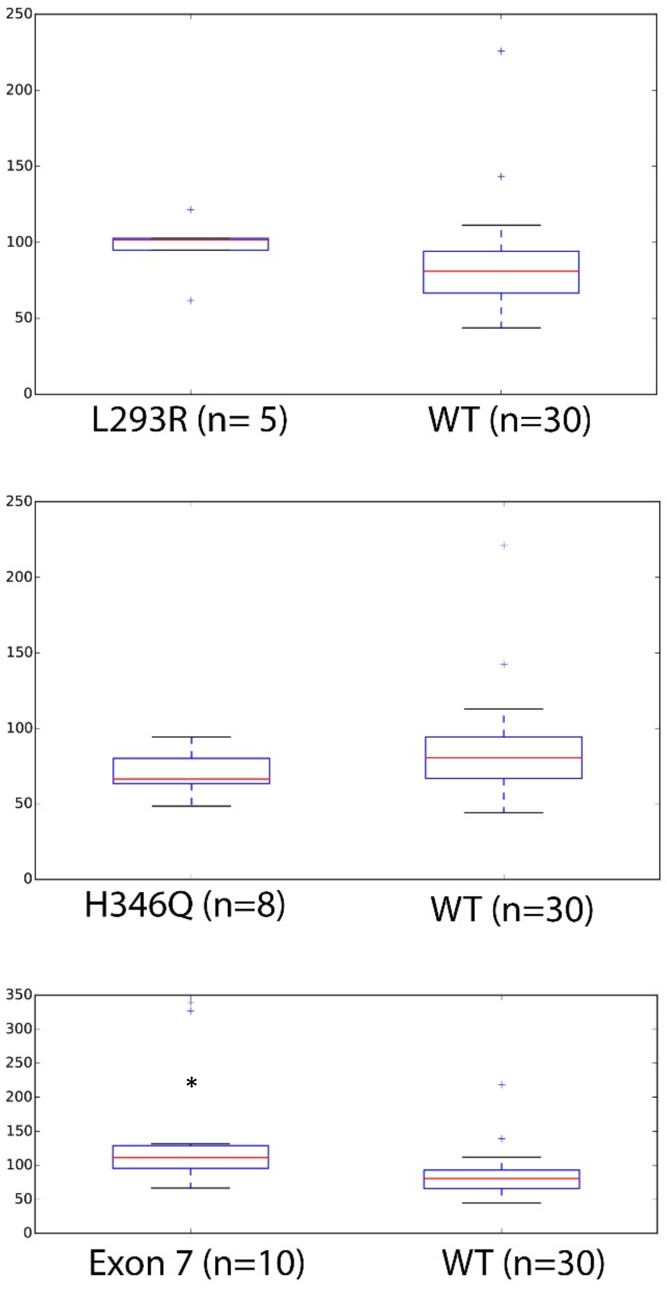

Alpha diversity comparisons were conducted using a two-sample t test and nonparametric Monte Carlo permutations (n = 999) within QIIME-1.9.1 to compare average observed species richness measures between cohorts after conducting multiple rarefactions to remove any biases associated with sequencing depth. Exon7 vs WT, *P<0.05.
